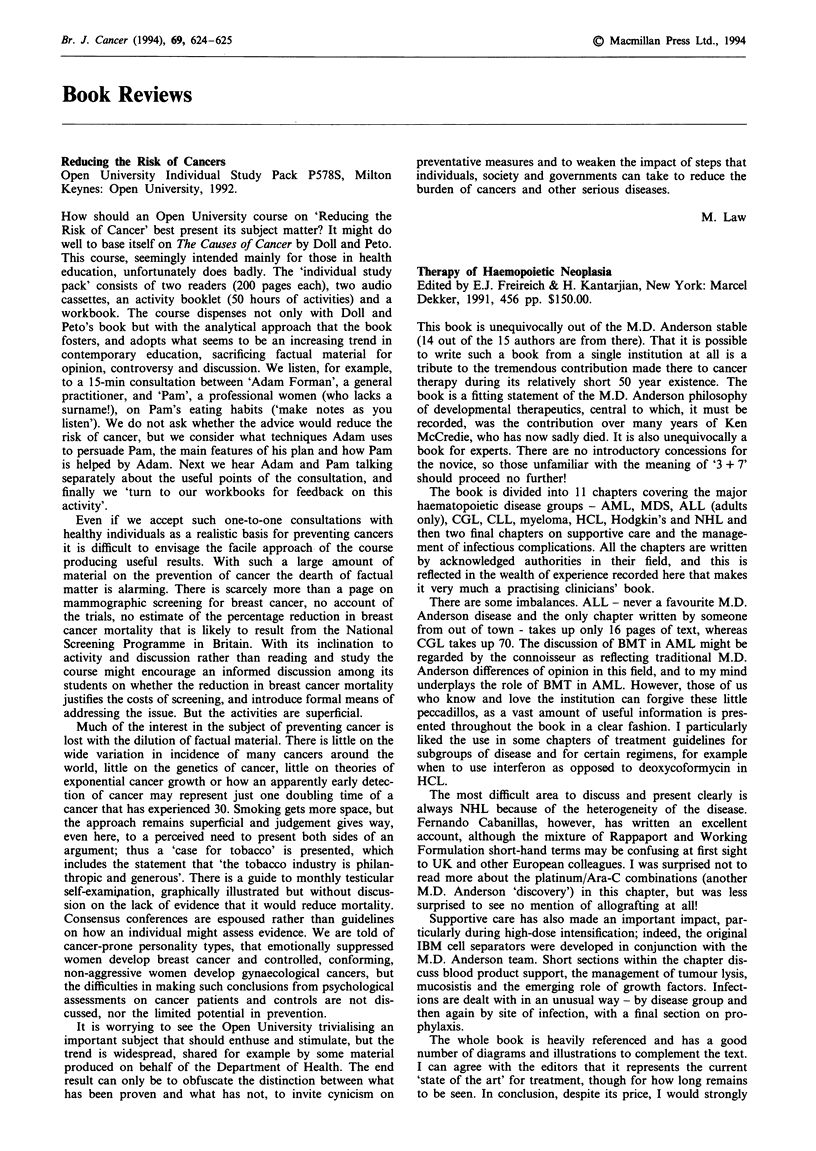# Reducing the Risk of Cancers

**Published:** 1994-03

**Authors:** M. Law


 © Macmillan Press Ltd., 1994

### Book Reviews

**Reducing the Risk of Cancers**
Open University Individual Study Pack P578S, Milton Keynes: Open University, 1992.

How should an Open University course on 'Reducing the Risk of Cancer' best present its subject matter? It might do well to base itself on *The Causes of Cancer* by Doll and Peto. This course, seemingly intended mainly for those in health education, unfortunately does badly. The 'individual study pack' consists of two readers (200 pages each), two audio cassettes, an activity booklet (50 hours of activities) and a workbook. The course dispenses not only with Doll and Peto's book but with the analytical approach that the book fosters, and adopts what seems to be an increasing trend in contemporary education, sacrificing factual material for opinion, controversy and discussion. We listen, for example, to a 15-min consultation between 'Adam Forman', a general practitioner, and 'Pam', a professional women (who lacks a surname!), on Pam's eating habits ('make notes as you listen'). We do not ask whether the advice would reduce the risk of cancer, but we consider what techniques Adam uses to persuade Pam, the main features of his plan and how Pam is helped by Adam. Next we hear Adam and Pam talking separately about the useful points of the consultation, and finally we 'turn to our workbooks for feedback on this activity'.

Even if we accept such one-to-one consultations with healthy individuals as a realistic basis for preventing cancers it is difficult to envisage the facile approach of the course producing useful results. With such a large amount of material on the prevention of cancer the dearth of factual matter is alarming. There is scarcely more than a page on mammographic screening for breast cancer, no account of the trials, no estimate of the percentage reduction in breast cancer mortality that is likely to result from the National Screening Programme in Britain. With its inclination to activity and discussion rather than reading and study the course might encourage an informed discussion among its students on whether the reduction in breast cancer mortality justifies the costs of screening, and introduce formal means of addressing the issue. But the activities are superficial.

Much of the interest in the subject of preventing cancer is lost with the dilution of factual material. There is little on the wide variation in incidence of many cancers around the world, little on the genetics of cancer, little on theories of exponential cancer growth or how an apparently early detection of cancer may represent just one doubling time of a cancer that has experienced 30. Smoking gets more space, but the approach remains superficial and judgement gives way, even here, to a perceived need to present both sides of an argument; thus a 'case for tobacco' is presented, which includes the statement that 'the tobacco industry is philanthropic and generous'. There is a guide to monthly testicular self-examination, graphically illustrated but without discussion on the lack of evidence that it would reduce mortality. Consensus conferences are espoused rather than guidelines on how an individual might assess evidence. We are told of cancer-prone personality types, that emotionally suppressed women develop breast cancer and controlled, conforming, non-aggressive women develop gynaecological cancers, but the difficulties in making such conclusions from psychological assessments on cancer patients and controls are not discussed, nor the limited potential in prevention.

It is worrying to see the Open University trivialising an important subject that should enthuse and stimulate, but the trend is widespread, shared for example by some material produced on behalf of the Department of Health. The end result can only be to obfuscate the distinction between what has been proven and what has not, to invite cynicism on preventative measures and to weaken the impact of steps that individuals, society and governments can take to reduce the burden of cancers and other serious diseases.

M. Law